# *Klebsiella pneumoniae* Carbapenemase, Canada

**DOI:** 10.3201/eid1505.081536

**Published:** 2009-05

**Authors:** Dylan R. Pillai, Roberto Melano, Prasad Rawte, Stephen Lo, Nathalie Tijet, Milan Fuksa, Nancy Roda, David J. Farrell, Sigmund Krajden

**Affiliations:** Ontario Agency for Health Protection and Promotion, Toronto, Ontario, Canada (D.R. Pillai, R. Melano, P. Rawte, S. Lo, N. Tijet, D.J. Farrell); University of Toronto, Toronto (D.R. Pillai, R. Melano, S. Krajden); University Health Network, Toronto (D.R. Pillai); St. Joseph’s Health Centre, Toronto (M. Fuksa, N. Roda, S. Krajden)

**Keywords:** Antimicrobial resistance, bacteria, respiratory infections, Gram negative, carbapenemase, Canada, letter

**To the Editor:** Carbapenems are used to treat life-threatening infections caused by extremely drug-resistant gram-negative pathogens; these drugs represent the last line of defense in the antimicrobial drug armamentarium against serious or invasive infection (*1*). The rapid global spread of *Klebsiella pneumoniae* that produces *K. pneumoniae* carbapenemase (KPC), especially in the northeastern United States (e.g., New York state), is of major concern (*2*,*3*). KPC β-lactamases belong to the family of serine carbapenemases and are usually found in *K. pneumoniae* and *Escherichia coli.* KPC hydrolyzes β-lactam agents, thereby reducing their action. KPC activity has been reported, albeit less frequently, in other family *Enterobacteriaceae* (*K. oxytoca*, *Enterobacter* spp., *Salmonella* spp., *Citrobacter freundii*, and *Serratia* spp.) as well as in *Pseudomonas aeruginosa* (*1*).

The *bla*_KPC_ genes have been identified on conjugative plasmids and pose an infection control problem because plasmids could theoretically be transmitted from one species to another (*4*). The few therapeutic options for treating infections caused by organisms containing these β-lactamases are aminoglycosides, glycylcyclines, polymyxins, or combinations (*1*). A major concern is that routine susceptibility testing methods based on existing breakpoints can falsely identify KPC producers as susceptible to carbapenems. Such results pose the potential risk for increased illness and death, longer hospital stays, and nosocomial spread of infection.

In 2008, the Public Health Laboratory in Toronto received clinical isolates of *K. pneumoniae* from urine and sputum of 1 patient. The hospital laboratory had forwarded the isolates to the Public Health Laboratory because they were possible KPC producers. The patient was a 73-year-old man with a history of emphysema and hypertension, seen at a tertiary care hospital in the Toronto area, 80 miles from the New York state border, for a laparoscopic right radical nephrectomy because of hypernephroma. He had no risk factors for acquisition of KPC producers, e.g., travel to the United States or prior carbapenem exposure.

Susceptibility testing of *K. pneumoniae* was performed by the agar dilution method, using breakpoints set by the Clinical and Laboratory Standards Institute (*5*,*6*). The sputum isolate (7315) was susceptible to meropenem (MIC 4 μg/mL), and the urine isolate (7184) was intermediately susceptible (MIC 8 μg/mL). The *K. pneumoniae* isolates were screened for extended-spectrum β-lactamases (ESBLs) and AmpC production according to Ontario guidelines (*7*).

Briefly, to screen for ESBL enzymatic activity, a double-disk diffusion method was used: a clavulanic acid–containing disk was placed adjacent to a disk containing one of several cephalosporins such as ceftazidime and cefotaxime. Enhanced killing of the organism in the area between the drug with and without clavulanate indicates ESBL. Cefoxitin resistance (zone <17 mm) indicates AmpC-like β-lactamase activity. In addition, testing for ESBL/AmpC was performed according to Clinical and Laboratory Standards Institute guidelines (*6*). When the screening result for ESBL or AmpC is positive, the clinical laboratory issues a warning that no β-lactam except carbapenems can effectively treat this infection. The Table summarizes results of initial susceptibility testing and supplementary laboratory testing for KPC.

**Table:** Results of initial susceptibility and supplementary testing for *Klebsiella pneumonia*e carbapenemase in urine and sputum samples from 73-year-old man, Canada*

Isolate	MIC, µg/mL†							Disk diffusion results, mm					Initial report‡	Final report§
	AMP	FOX	CIP	GEN	CTRX	MEM		FOX	CAZ	CAC	CTX	CTC		
7184	>16	>16	>2	8	>32	8		16	0	14	13	15	AmpC/ESBL	KPC
7315	>16	>16	>2	8	>32	4		0	0	8	13	15	AmpC/ESBL	KPC

*AMP, ampicillin; FOX, cefoxitin; CIP, ciprofloxacin; GEN, gentamicin; CTRX, ceftriaxone; MEM, meropenem; CAZ, ceftazidime; CAC, ceftazidime-clavulanic acid; CTX, cefotaxime; CTC, cefotaxime-clavulanic acid; ESBL, extended-spectrum β-lactamase; KPC, *Klebsiella pneumoniae* carbapenemase. †MIC values for clinical isolates 7184 (urine) and 7315 (sputum) were obtained by using agar macrodilution. ‡Initial screening for ESBL or AmpC β-lactamase activity, performed by Kirby Bauer disk diffusion according to Clinical Laboratory Standards Institute guidelines (*6*,*7*), suggested ESBL or AmpC β-lactamase activity. §Supplementary modified Hodge test; PCR (specific for *bla*_KPC_ family), and DNA sequencing confirmed the presence of KPC activity due to *bla*_KPC-2_.

The initial result was consistent with a possible AmpC/ESBL producer for the sputum and urine isolates (*6*,*7*). However, because the patient responded poorly to empiric vancomycin and imipenem therapy and because of the elevated MIC to meropenem for isolate 7184, further laboratory testing was conducted to rule out the possibility of carbapenemase activity.

The modified Hodge test is a phenotypic test proposed to confirm the presence of carbapenemase activity such as KPC in *K. pneumoniae* and *E. coli* (*8*). Universal primers for *bla*_KPC_ family, Uni-KPC-F (5′-ATGTCACTGTATCGCCGTCT-3′) and -R (5′-TTACTGCCCGTTGACGCCC-3′), were used for the entire 882-bp coding sequence. Amplicons were bidirectionally sequenced by using the BigDye Terminators method and a 3130xl Genetic Analyzer (Applied Biosystems, Foster City, CA, USA) and primers Uni-KPC-F and -R. Multiple nucleotide and protein sequence alignments were performed with the ClustalW2 software (www.ebi.ac.uk/Tools/clustalw2/index.html). To aid the clinician, an Etest method was used to measure the MIC of this KPC-producing *K. pneumoniae* isolate to colistin (0.5 μg/mL) and tigecycline (2.0 μg/mL). However, before this information could be used, the patient had died of respiratory failure, presumably caused by *K. pneumoniae*. Infection control measures and laboratory screening were undertaken in the hospital to limit transmission to other patients.

This report shows that KPC-producing organisms such as *K. pneumoniae* may pose a major risk for clinical disease and a challenge for infection control if they were to spread to other hospitals in Canada. Current testing algorithms focus on ESBL- and AmpC-producing gram-negative bacteria, which may not detect KPC-producer strains. We suggest that reference laboratories validate a screening method coupled with confirmatory phenotypic assay for carbapenemase activity for suspected organisms, especially *K. pneumoniae* and *E. coli*. Our in-house validation studies confirm that use of the ertapenem disk followed by the modified Hodge test to confirm carbapenemase activity may be effective (D.R. Pillai et al., unpub. data). Public health officials should be aware that this report further expands the international distribution of KPC-producing *K. pneumoniae*.
